# Co-Creation in the Development of Digital Therapeutics: A Narrative Review

**DOI:** 10.3390/ijerph21121589

**Published:** 2024-11-28

**Authors:** Inês Mimoso, Teodora Figueiredo, Luís Midão, Joana Carrilho, Diogo Videira Henriques, Sara Alves, Natália Duarte, Maria João Bessa, David Facal, Alba Felpete, José María Fidalgo, Elísio Costa

**Affiliations:** 1 CINTESIS@RISE, Biochemistry Lab, Faculty of Pharmacy, University of Porto, 4050-313 Porto, Portugal; ifmimoso@ff.up.pt (I.M.); tgfigueiredo@ff.up.pt (T.F.); lmidao@ff.up.pt (L.M.); jcarrilho@ff.up.pt (J.C.);; 2 Department of Biological Sciences, Faculty of Pharmacy, University of Porto, 4050-313 Porto, Portugal; 3 Porto4Ageing—Competences Centre on Active and Healthy Ageing, Faculty of Pharmacy, University of Porto, 4050-313 Porto, Portugal; 4 CINTESIS@RISE, Instituto de Ciências Biomédicas Abel Salazar, University of Porto, 4050-313 Porto, Portugal; 5 Santa Casa da Misericórdia de Riba D’Ave/CIDIFAD—Centro de Investigação, Diagnóstico, Formação e Acompanhamento das Demências, 4765-220 Riba D’Ave, Portugal; 6 UPTEC-Science and Technology Park, University of Porto, 4200-135 Porto, Portugal; 7 Department of Developmental Psychology, University of Santiago de Compostela, 15705 Santiago de Compostela, Spain; david.facal@usc.es (D.F.);; 8 ACIS-Agencia Gallega para la Gestión del Conocimiento en Salud, 15707 Santiago de Compostela, Spain

**Keywords:** digital therapeutics, co-creation, participatory design, stakeholder involvement

## Abstract

Digital therapeutics (DTx) are transforming healthcare delivery through personalised, evidence-based interventions that offer a cost-effective approach to health management. However, their widespread adoption faces significant barriers including privacy concerns, usability issues, and integration challenges within healthcare systems. This review assesses the current evidence on DTx, with a particular focus on the role of co-creation in enhancing design and usability. A narrative review was conducted to identify studies exploring co-creation in DTx development. Three studies were selected for in-depth analysis, demonstrating that co-creation processes significantly improve the usability and effectiveness of DTx interventions. Findings underscore challenges in DTx implementation, including complex regulatory processes, digital inequality, high development costs, and difficulties in integrating with existing healthcare systems. Despite the existence of discrete examples of co-creation in DTx and its acknowledged value in the healthcare domain, systematic research in this field remains markedly limited. Future studies should prioritise establishing best practises for co-creation, with particular emphasis on methods to enhance data privacy and security, standardisation protocols, and patient engagement strategies to optimise DTx adoption and effectiveness. This review contributes to the growing body of literature on DTx by highlighting the potential of co-creation while also identifying critical areas for future research.

## 1. Introduction

The rapid evolution of digital technologies is making far-reaching changes in healthcare delivery with digital health, digital therapeutics (DTx), and mobile health (mHealth) playing crucial roles in enhancing patient care and health management. Mobile technologies such as smartphones, tablets, and smartwatches have become widespread in modern society, making them highly versatile tools for several applications, including healthcare [1]. Over the past decade, digital health has transformed health monitoring and well-being practises from activities traditionally confined to hospitals and clinics to more accessible remote solutions through various digital devices, particularly smartphone applications and smartwatches. The COVID-19 pandemic has further accelerated this shift towards digital healthcare [1,2,3,4].

“Digital Health” covers technologies (e.g., telehealth, wearable devices, mHealth apps) that actively engage patients in managing their health and well-being. As healthcare increasingly embraces digital solutions and patients become more digitally literate, they are now more empowered to take control of their health than ever before [2]. The rise of “e-patients,” individuals who are equipped, enabled, empowered, and engaged in their health and healthcare decisions through digital technologies, pinpoints the growing demand for digital health tools [2]. This digital transformation is expected to revolutionise healthcare, providing innovative solutions that promote accessibility and efficiency, while improving patients’ health status, quality of life, and overall well-being. It will reshape healthcare, making it more responsive and integrated into everyday life, benefiting both patients and healthcare organisations [1]. While the digitalisation of healthcare continues to expand rapidly, it is unlikely to ever fully replace the relationship between patients and healthcare professionals. Instead, it functions as a complementary tool, providing a supportive platform to enhance the delivery of care [5,6].

DTx health software is intended to treat or alleviate a disease/condition, offer therapeutic interventions to patients delivered through high-quality software programmes, and is set to revolutionise healthcare [1,7]. DTx differs from mHealth in its purpose, scope, and regulatory requirements. Conversely, DTx also presents challenges, including the need for clinical validation, complex regulatory pathways, and lower widespread adoption among healthcare providers and patients [8]. Additionally, challenges such as digital access inequality and privacy concerns hinder equitable access. These challenges will only be overcome with more research and practical implementation. However, to address challenges such as lower user engagement and adherence, usability and accessibility issues, a lack of custom and personalised solutions, low rates of trust and, finally, difficulties in integration into healthcare systems, co-creation can be a promising approach [8,9]. Involving end-users and stakeholders in the design and development process will lead to more effective and user-friendly DTx solutions that are better aligned with the needs and preferences of their target audience [10,11,12].

Despite the benefits, the integration of co-creation in the development of DTx is still in its early stages. While evidence supports the use and effectiveness of co-creation in mHealth—a wide range of applications designed to promote health and wellness through mobile devices [9,10,13,14]—there is a scarcity of similar studies focusing specifically on DTx [15,16,17]. This underscores the need for more research and documentation on the use and benefits of co-creation in the development of DTx, particularly in the early stages of development.

The purpose of this article is to review the current evidence on the use and application of DTx, calling attention to the importance of co-design and participatory design in its development. By examining existing practises and identifying the challenges and opportunities, this review seeks to provide a comprehensive overview of the role of user engagement in designing DTx interventions. The amount of the literature specifically addressing this topic is limited, with only three articles found on the use of co-creation in DTx development. Overall, there is a notable scarcity of published research on co-creation in the context of digital therapeutics.

## 2. Methods

The present narrative review was conducted to synthesise and analyse the relevant literature on digital therapeutics (DTx) and the role of co-creation in their development and adoption. It does not aim to be a systematic syntheses that answer a specific, highly focused question; instead, it offers carefully thought out and rigorous interpretations of what DTx are; what they are used for; evaluation, regulation, and approval; and it especially focuses on the use of co-creation methodologies to design and develop more tailored DTx solutions. This type of review was selected as it offers a critical synthesis of the literature.

A search for studies was carried out across academic databases such as PubMed, Scopus, and IEEE Xplore, focusing on articles published between 2019 and 2024 and written in the English language. A combination of keywords was used to identify relevant articles. The search terms included the following: (“Digital Therapeutics” OR DTx OR “digital health” OR “digital health interventions” OR “mHealth Interventions” OR “eTherapeutics” OR “Medical Apps” OR “Therapeutic Software” OR “therapeutic interventions” OR “medical software” OR “behavioral interventions” OR “digital treatment”) AND (co-design OR co-creation OR “participatory design” OR “User-Centered Design” OR “End-User Involvement” OR “human-centred design” OR “Design Thinking” OR “focus group” OR “ethnographic research” OR “human-factors engineering” OR “user engagement” OR “patient-centered care”). A systematic search strategy with rigid selection criteria was not applied; instead, studies were selected based on their relevance and quality. Studies that discussed the application of co-creation in DTx development, as well as challenges and barriers to adoption, were included. No restrictions were placed on the type of study (quantitative, qualitative, or mixed-methods), allowing for a broader approach. The analysis was descriptive, identifying emerging patterns in the findings, key barriers to DTx adoption, and the effectiveness of co-creation methodologies. The authors explicitly recognise that they may not include all the relevant literature on the topic. The literature was critically analysed, highlighting existing gaps and future research directions.

## 3. Narrative

### 3.1. Digital Therapeutics

According to the technical report, ISO/TR 11147: Health informatics—Personalised digital health—Digital therapeutics health software systems (2023), a digital therapeutic is a “health software intended to treat or alleviate a disease, disorder, condition, or injury by generating and delivering a medical intervention that has a demonstrable positive therapeutic impact on a patient’s health” [18]. In other words, DTx offers therapeutic interventions to patients delivered through high-quality software programmes [2]. DTx can be used independently or in addition to other therapies, medical devices or pharmaceutical products and can be used with or without the supervision of a healthcare practitioner [19].

Therefore, for a programme/mobile application to be considered a DTx, it must meet the following criteria [20,21]:

High-quality software programmes: DTx are a type of Software as a Medical Device (SaMD), which, since they are considered independent medical devices, do not require European Medicines Agency (EMA)- or Food and Drug Administration (FDA)-regulated hardware, only the user’s smartphones or tablet [22,23,24]. DTx are often coupled with artificial intelligence (AI) and machine learning systems [25].

Evidence-based: Medical evidence must be obtained, which requires publishing clinical trial outcomes in peer-reviewed journals and having them assessed and approved by regulatory bodies (see Section 3.2). It involves collecting and analysing real-world evidence and device performance data. Clinical evidence is often sourced from randomised clinical trials (RCTs), mirroring the methodology employed for traditional pharmaceuticals and medical devices [24].

Make a claim to prevent, manage or treat a medical disease, disorder, condition, or injury: the primary goal of DTx is to deliver treatment. These products offer therapeutic benefits similar to those provided by other traditional therapies, such as medications or medical devices [26].

DTx can be particularly beneficial in the context of chronic disease management, where continuous monitoring and personalised interventions can significantly improve patients’ outcomes [2]. For instance, DTx solutions are being developed and used for conditions such as diabetes, mental disorders, and cardiovascular disease, demonstrating their potential to enhance treatment efficacy and patient engagement [2].

Since it may not always be easy to recognise whether a particular application/programme is a DTx or just mHealth, the Digital Therapeutics Alliance (DTA), an international organisation dedicated to the understanding and adoption of DTx, in its website provides a flowchart to help users, clinicians, and the general audience distinguish one from another [20].

The growth in the global market value of DTx is notorious, standing at 1.8 billion in 2018, 5.09 billion in 2022, and is expected to grow to 7.1 billion in 2025 [2,19,27]. These data demonstrate the rising importance of DTx in the healthcare sector.

#### 3.1.1. Digital Health vs. Digital Medicine vs. DTx

Digital health—everything digital that relates to health—is an umbrella term that includes everything from mHealth applications, electronic health records (EHRs), telehealth, digital medicine, DTx, smart devices, sensors, and wearables. Digital health does not require clinical evidence or regulatory oversight [28].

**Figure 1:**
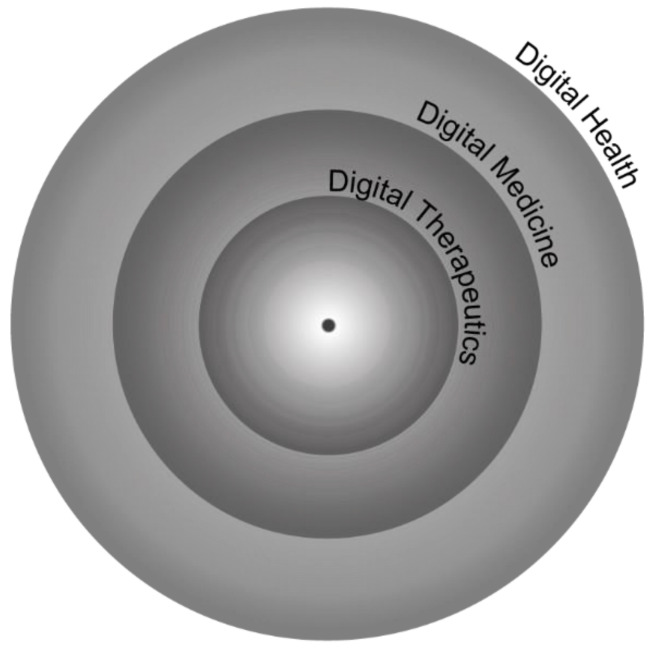
Digital therapeutics as a subset of digital health—DTx is part of a larger system, hinting at the interconnected relationships with other digital health technologies. Adapted from Ahlqvist J, Kalliola M. How can digital therapeutics help Europe? Institutional Report. Sitra; 2021. Available from https://www.sitra.fi/en/publications/how-can-digital-therapeutics-help-europe/ (accessed on 30 July 2024).

Digital medicine, a more specific subset within digital health, focuses on technologies that deliver evidence-based therapeutic interventions. These interventions require rigorous clinical validation to ensure their safety and efficacy [28]. Conversely, DTx represent the forefront of digital health, distinguished by tools that are both clinically validated and demonstrate tangible outcomes in real-world settings. Digital medicine, positioned between digital health and DTx (Figure 1), demands clinical evidence but does not necessarily require proof of effectiveness in real-world applications [28].

Each digital health sub-category carries different claims, risks, clinical evidence requirements, and regulatory oversight, yet all contribute uniquely to modern healthcare practises [2,8,24].

#### 3.1.2. DTx vs. mHealth

**Figure 2:**
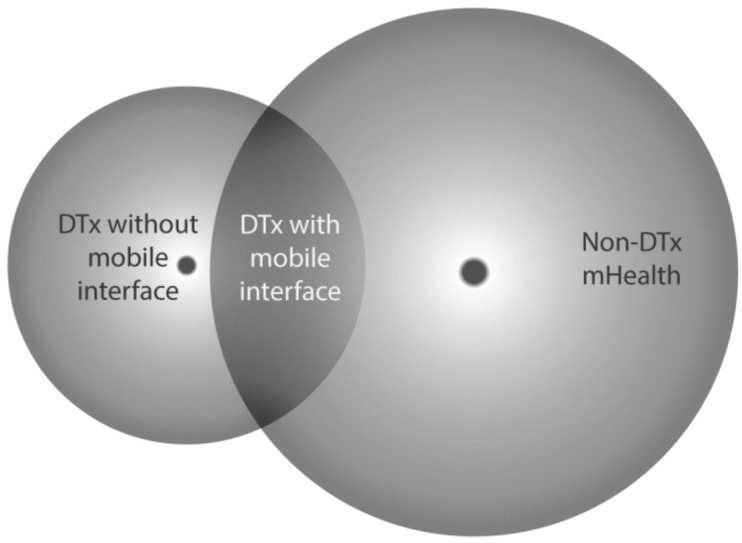
Digital therapeutics as a subset of mHealth. Adapted from Ahlqvist J, Kalliola M. How can digital therapeutics help Europe? Institutional Report. Sitra; 2021. Available from https://www.sitra.fi/en/publications/how-can-digital-therapeutics-help-europe/ (accessed on 30 July 2024).

While digital therapeutics (DTx) fall under the broader category of mobile health (mHealth), not all mHealth solutions qualify as DTx (Figure 2). DTx are a specific category that only includes evidence-based therapeutic interventions that prevent, manage, or treat a disorder or disease. In contrast, many mHealth applications focus on general health and wellness, such as tracking fitness or promoting healthy behaviours, without providing the medical interventions that are characteristic of DTx. As shown in Figure 1, DTx are a distinct subset within the field of digital health and although DTx are a subset of mHealth, not all mHealth solutions qualify as DTx [8].

#### 3.1.3. Evaluation, Regulation, and Approval

For DTx to be introduced into the market and made available to the public, they must have approval and/or market authorisation from a regulatory body. Regulatory requirements established by agencies like the European Union’s Medical Device Regulation (MDR) and EMA in Europe and the FDA in the United States of America provide the standards for validating DTx. These standards are designed to safeguard patient welfare and ensure that the evidence supporting these therapies meets established criteria for both efficacy and safety [8].

Within the EU, no specific legal regulation exists on DTx. The clinical investigation and sale of medical devices for humans falls within Regulation (EU) 2017/745 [29]. SaMD products are governed by the MDR, which outlines the procedures for the design, development, and validation of software intended for medical purposes for the European market. However, compliance with the MDR alone does not guarantee the general reimbursement of medical devices and neither details the methods for validating performance, safety, accuracy, and usability [22,30]. The presence of the CE mark on a medical application also does not guarantee that it adheres to best practises (e.g., failure to include user-centred design principles in the development process, lack of comprehensive usability testing with target populations) or has been thoroughly tested for accuracy or clinical benefits (e.g., insufficient clinical trial data demonstrating efficacy compared to existing treatments). These aspects can only be confirmed by the EMA and relevant national authorities [30].

On a national level, Germany was a pioneer in producing regulations for SaMD products, taking a leading role in reimbursement policies with the DiGA Fast-Track system—a structured framework for the reimbursement of prescribable health applications on a large scale that also regulates specific requirements for the use of DTx [22]. In this country, the surveillance of approved DTx relies on active reporting from users and healthcare providers, with no adverse effects reported in trials [19]. France is also moving forward to implement a similar legal act like the German DiGA Fast-Track system [29]. Currently, Portugal does not have any system for reimbursing DTx or mHealth apps.

Most clinical evaluations of DTx have employed clinical trials, aligning with the standards used for assessing drugs and medical devices. However, given that DTx are digital health products, there is a need for more active patient engagement assessment than traditional drugs, with the user interface playing a crucial role [8,24]. On the other hand, the impact of product updates on approval processes should also be considered since iterative changes and regular updates to software are very common in any digital product [8]. Some potential contraindications, like blue light affecting sleep quality and increased anxiety or stress due to constant health monitoring, remain unaddressed in the usage instructions [19]. It would be important to ensure the safety of use in any DTx available in the market, not only the evidence of patient outcomes.

#### 3.1.4. Major DTx Products and Companies

In this review, there were identified 20 DTx currently available on the market, as shown in Table 1. These products conform to the definition of digital therapeutics and align with the established DTA Core Principles [7]. The DTx included in Table 1 are classified according to whether or not they require a doctor’s prescription; the company that developed the technology; the therapeutic areas in which they are used; and their approval status in the various countries.

#### 3.1.5. DTx Advantages and Disadvantages

DTx offers significant advantages by giving patients a deeper understanding of their diseases or conditions. This knowledge leads to greater involvement in the decision-making and treatment processes, empowering patients to take an active role in their healthcare. Self-management, another characteristic promoted by DTx, allows individuals to monitor and manage their health needs independently, leading to improved health outcomes and enhanced patient autonomy [9].

Diseases, like cancer and diabetes, can lead to a wide range of cognitive, psychological, physical, and social challenges that many survivors find extremely debilitating. Often, there is a lack of adequate post-treatment support, especially for those living with disabilities. Within this framework, digital solutions, especially DTx, can be an advantage. By providing accessible, personalised, and evidence-based interventions, DTx can enhance the quality of life for survivors, addressing the multifaceted challenges they face and offering continuous support in the comfort of their homes [1]. DTx are therefore not only a means of treating diseases and conditions but also play a crucial role in mitigating consequences and side effects. DTx can ensure that patients receive full support, improving overall health outcomes and quality of life by not only targeting the disease itself but also managing the broader impact it may have on daily living.

As mentioned earlier, in some countries, DTx, when prescribed by physicians, can qualify for reimbursement from public and private health payers, much like traditional medications [30].

On the contrary, the adoption and use of DTx also face barriers and constraints. Privacy concerns are present, as both patients and clinicians worry about the security of sensitive health information. Usability issues can hinder patient engagement and effectiveness if the technology is not user-friendly. Additionally, bugs and other quality issues may arise, and digital knowledge and skills are needed to effectively engage with technology [30]. Furthermore, there is still a perception among patients and healthcare providers that health-related technology can be unnecessary, which decreases the acceptance and integration of DTx into standard care practises. These obstacles must be addressed to fully realise the potential benefits of DTx [9].

Doubts are raised about the security of DTx, on whether developers reflect on adverse effects and risks. Knowledge about the negative consequences still needs to be improved [19]. Denecke et al. propose a Risk Assessment Canvas for DTx aiming to support critical reflection on what should be considered when developing and releasing DTx into the market, and during the prescribing and use of these apps.

While DTx offer significant potential, their practical implementation faces numerous challenges that extend beyond initial adoption barriers. Regulatory processes are demanding, requiring extensive clinical validation and compliance with standards set by regulatory bodies [8]. The lack of standardised regulatory frameworks across regions further complicates the global scalability of DTx solutions [31]. Digital inequality persists in many regions, limiting the reach of DTx to populations with reliable internet access and modern devices [32]. Additionally, the high cost of development and maintenance poses challenges to creating financially sustainable models that remain accessible to patients [31]. Integration into healthcare systems remains a critical hurdle, as many DTx lack interoperability with existing clinical workflows, leading to resistance from healthcare professionals [28]. Addressing these multifaceted challenges will require a concerted effort from developers, regulators, and healthcare stakeholders to ensure that the full potential of DTx can be realised in practise.

#### 3.1.6. User Perceptions and Experiences

Recently, a cross-sectional survey was conducted to explore end-user engagement with self-guided digital therapeutics for mental health management, involving 211 participants. The survey found that easily applicable content is crucial for engagement, while content that could trigger psychological distress should be avoided. Key facilitators of engagement included digestible content, assurance of confidentiality, and appealing design. In contrast, barriers included lack of time, forgetfulness, and concerns about efficacy. Additionally, the diverse user perspectives emphasise the need to co-design engagement strategies for digital interventions with individuals who have lived experiences [33].

Another study on digital therapeutics for insomnia identified key user concerns, including cost-effectiveness, evidence of efficacy, and privacy regarding personal data. Users also expressed demands for convenient use, reduced social stigma associated with DTx, compatibility with other healthcare systems, and better communication with healthcare providers through DTx platforms. The study emphasised that tailored approaches, which take patient characteristics into account, are essential for the broader adoption of these technologies [34].

Bally, E. et al., in a study that, although not specific to DTx, evaluates the perspectives of stroke patients on technological support and self-management solutions, found that co-design methodologies like semi-structured interviews revealed mixed attitudes regarding the use of technological solutions. Most participants recognised the value of having access to relevant health information. However, some participants expressed a preference for in-person contact with healthcare professionals over receiving care through technological or digital means [9].

In the same study, participants, when questioned about usability issues, highlighted the importance of adapting the user experience to older adults to ensure the acceptance of these technologies. They emphasised that technologies should be designed to be easy to use, so intuitive that users can operate them without consciously thinking about how to use them. Typing functions were perceived as more complicated, and it was suggested that the products should be compatible with multiple devices, such as tablets and computers, in addition to smartphones. Participants also expressed a desire for interfaces that could be easily customised to meet their individual needs and difficulties. Another significant concern was the challenge of using apps after they have been updated or improved; substantial changes to the interface can greatly hinder understanding for older users [9].

#### 3.1.7. Healthcare Professional’s Perceptions and Experiences

In the AMCP multidisciplinary stakeholder forum “Digital Therapeutics: What Are They and Where Do They Fit in Pharmacy and Medical Benefits?”, healthcare professionals highlighted an urgent need for expanded educational activities on DTx. Currently, there are few education opportunities about DTx, and these therapies are not typically included in training curricula. Moreover, DTx that demonstrate clinical effectiveness, especially comparative effectiveness, could be included in clinical practise guidelines to promote widespread use. Data usage and security also pose challenges. While DTx collect a lot of patient data, integrating these data into clinical practise can be difficult. Technical issues also arise with integrating DTx data into electronic health records, and requirements may differ based on whether the DTx is prescription or non-prescription [8].

A related study on physicians’ acceptance of DTx found that adoption is strongly influenced by both personal and institutional factors [35]. On a personal level, two key elements emerged: the perceived usefulness and ease of use of DTx. Physicians are more likely to incorporate DTx into their practise when they believe it will enhance efficiency and patient care [35]. This finding aligns with previous studies [8], underscoring the importance of demonstrating the clinical effectiveness of DTx to drive adoption. Regarding institutional factors, the study highlighted that support from hospitals and healthcare institutions—such as clear guidelines for integration, training programmes, and solutions to regulatory or reimbursement issues—plays a critical role. Peer influence also contributes significantly, as seeing colleagues successfully use DTx encourages broader acceptance and implementation among physicians [35].

### 3.2. Co-Creation: Co-Design

Engaging end-users, such as patients and healthcare providers, at every stage of designing and implementing health technologies is crucial for their success. By fostering collaboration, a process referred to as co-creation, these solutions can achieve greater societal acceptance, improved usability, and higher overall quality [36]. Including users in the design process is also becoming the standard practise, with health research centres acknowledging its importance and several research grant applications requiring user involvement [10]. This reflects the growing recognition of its value in creating effective and user-friendly solutions. Involving users in the development process ensures that the final product or service aligns with their actual requirements and preferences. User participation not only enhances functionality and usability but also fosters a sense of ownership and satisfaction, leading to greater acceptance and engagement with the product [37]. Furthermore, by incorporating user feedback, developers can identify and mitigate potential safety issues early in the design process, thereby creating safer healthcare solutions [12].

While user-centred design focuses on users and their needs throughout the stages of the design process, co-design, or participatory design, takes it one step further by fostering direct collaboration between designers/developers and users during the entire design process [11,13,38,39]. This approach is valuable in the development of health-related applications (including DTx), as it ensures that solutions are shaped by the real experiences and needs of end users and stakeholders [10,11]. Designing alongside the users also helps to “humanise” technologies, which are often criticised for being overly structured, rigid, and unresponsive [11]. Additionally, the literature argues that co-creation helps ensure that DTx solutions are sustainable, scalable, and aligned with end-users preferences, enhancing their effectiveness and safety [37].

As Laurisz et al. mention, although the company designs and supplies the product itself, its value emerges through interaction with the consumer [36]. Co-design studies in the health field should involve patients, families, carers and friends, clinicians and health professionals, pharma companies, technology providers, practitioners with expertise in digital technologies—designers, engineers, technology developers—and, finally, policymakers and payers [11,30]. Co-design is also a continuous process that spans the entire lifecycle of products/services, beginning as soon as the initial idea is conceived, and should accompany the product throughout its development and use [11].

#### 3.2.1. Co-Design Activities

Co-design is highly collaborative—with users contributing ideas and making decisions alongside designers—and can use a variety of engaging techniques to fully integrate users’ perspectives. However, the evidence on best practises for meaningful co-design with patients in digital health research is still lacking [12]. Typically, researchers first undertake preparatory co-design activities, such as identifying the relevant end-user groups, outlining their roles and responsibilities, and managing their recruitment and engagement in the project or programme. Co-design activities should be tailored to the end-user groups involved. It is important to recognise that engaging and involving children or older adults in the co-creation process is fundamentally different from working with stakeholders such as regulators. Each group brings unique perspectives, needs, and constraints, which require tailored approaches to ensure meaningful participation [40].

User scenarios: A user scenario, also known as user flow, is a narrative that illustrates how a user will interact with a product or service in a particular situation. Crafting these scenarios involves pinpointing a specific context and understanding the user’s needs and attitudes. User scenarios can be complemented with visual elements such as drawings, photographs, or video clips that add a visual dimension to the narrative [41]. User scenarios are especially useful in the early stages of DTx development because they provide insights into real-world interactions with the technology. They can help identify potential usability issues and ensure the design meets user needs, improving the overall effectiveness and integration of the DTx and their future integration in daily life. User scenarios can only be considered co-design activities when they are created in collaboration with users.

Personas: A persona is a reference model that represents a specific user type with realistic characteristics—name, age, household composition, etc. These personas should reflect the needs, desires, habits, and cultural backgrounds of user groups. This activity is used to help the designer remember who they design for and get inspired by their specific life and challenges, which helps in understanding the diverse backgrounds and requirements [42]. Creating personas is beneficial for developing DTx because they offer a clear, detailed representation of target users, including their needs, preferences, and behaviours. This will lead the developer to more user-centred and effective DTx solutions tailored to real-world requirements. Personas can only be considered co-design activities when they are created in collaboration with users.

Ethnography: Ethnographic research is a qualitative methodology aimed at understanding the social interactions, behaviours, beliefs, and perceptions within groups, organisations, and communities. This approach provides rich insights into various cultures and subcultures by immersing the researcher in the same social space as the participants. Data collection typically involves participant observation, interviews, and focus groups, enabling researchers to gather rich, contextual insights that inform the design and bridge the gap between users and designers [43].

Despite its many advantages, ethnographic research presents challenges, such as being time- and resource-consuming. The researcher’s presence and subjective interpretation can introduce bias, and there are ethical considerations related to privacy and consent when involving participants in their natural settings. Nonetheless, the insights gained from ethnographic research make it a valuable tool for enhancing the design and implementation of interventions in complex real-world environments. The most relevant methods used in ethnographic studies are listed below:

Observation:

Direct observation: the researchers do not engage with the participant’s activities, assuming the role of eyewitnesses [43].

Participant observation: Requires the researcher to engage with the activities and routines of the people that are being observed. Used when one needs to learn more about the inner workings and the internal culture of a particular group or an organisation [43].

Interviews:

Structured interviews: Characterised by a “rigid” format and controlled circumstances, ensure that results are as consistent as possible. Each question follows a closed script, allowing minimal room for elaboration, resulting in immediate and spontaneous responses [43].

Semi-structured interviews: Follow a predetermined script but allow for more extensive responses and the inclusion of additional information. They accommodate unplanned questions, facilitating a more natural and dynamic dialogue between interviewer and interviewee [43].

In-depth interviews: Explore in depth a respondent’s point of view, experiences, feelings, and perspectives. This type of interview is open-ended and takes the form of a conversation [43].

Focus groups: guided group conversation to gather feedback on how people feel about issues taking into consideration other people’s feelings [43].

Ethnographic studies are valuable for developing DTx because they offer in-depth insights into users’ everyday environments and interactions. By immersing in users’ real-life contexts, researchers can uncover subtle, often overlooked aspects of user behaviour and needs. This helps in designing DTx that are more accurately aligned with users’ actual practises and challenges, leading to more effective solutions and giving the users and stakeholders a central role in the development of the solutions.

Surveys: A common form of quantitative research used to document individuals’ characteristics, opinions, attitudes, or past experiences. As one of the most used methods of quantitative research, they may seem straightforward, but designing effective surveys demands significant expertise [43].

Workshops: Workshops are collaborative and creative design sessions that bring users and other stakeholders into the design process. They are crucial for designing and developing digital health solutions, especially for vulnerable groups. Workshops are a good resource for developing DTx as they promote collaborative ideation and problem-solving among diverse stakeholders, including users, designers, and health professionals. Workshops also foster hands-on engagement with prototypes, allowing immediate testing and iteration based on participant input [43].

Usability studies: Usability studies evaluate how effectively users interact and understand digital products. In these activities, a researcher asks the participants to perform tasks using user interfaces. During this process, the researcher monitors the participant’s actions and gathers their feedback. Researchers can record user activity for later analysis [44]. Usability testing allows us to identify the design’s strengths and areas requiring improvement. Evidence suggests that digital health platforms often enhance the safety and quality of care [45]. However, some studies have identified unintended negative effects due to poor usability [46]. Designing digital health technologies is complex. Poor usability can lead to increased medical errors, higher costs, decreased efficiency, and user dissatisfaction [47]. Therefore, usability studies are crucial for developing DTx, as they help ensure that these technologies are user-friendly and integrate seamlessly into clinical practise. As Adler et al. showed, usability testing using the SUS questionnaire is a common effective method for evaluating the usability of mHealth apps [1]. The study of Jeong et al. analysing usability tests of DTx showed that the design of virtual agents significantly impacts both usability and therapeutic outcomes, recommending the need to consider agent-centric designs to optimise therapeutic efficacy and user engagement in DTx [48]. Another study exploring which usability assessment for digital therapeutics should be performed emphasised the need for a defined standardised approach to usability assessment in digital therapeutics. It discusses various methods for evaluating usability, highlighting that different assessments should be tailored to the specific contexts and users of digital therapeutics. However, specific further studies focused on usability testing for digital therapeutics are needed.

There are numerous ways to approach co-creation, with a variety of practical exercises that can be adapted to the activities listed. The choice of methods depends largely on the users involved, the specific conditions of the project, and the needs being addressed. This flexibility ensures that the co-creation process remains relevant and tailored to each unique context. By focusing on the relevant activities listed, researchers and developers can ensure a comprehensive understanding of user needs and create digital health solutions that are user-centric, inclusive, and effective. However, the development and validation of standardised processes for DTx should be promoted to ensure consistency, quality, and scalability across different projects and contexts.

#### 3.2.2. Benefits and Challenges Associated with the Use of Co-Creation in DTx Development

As mentioned, co-design plays a crucial role in engaging patients, caregivers, and healthcare professionals by allowing them to reflect on their experiences with a particular service or product and identify key areas for improvement. By incorporating direct input from end-users, co-design has the potential to significantly enhance the adoption and adherence to digital therapeutics, ultimately leading to more effective and user-centred healthcare solutions [9].

The development of a DTx platform involves several stages, including ideation, needs analysis, defining, prototyping, designing, testing, creating a marketing strategy, commercialisation, iteration, growth, evaluation, and maturity. Each phase is critical to ensure the clinical efficacy, user acceptance, and market success of the DTx [49]. The true potential of DTx can only be reached when patients are actively involved in both the development and use of these solutions. Digital therapeutics are only effective if users meaningfully engage with the product. This participatory approach ensures that DTx are tailored to meet the actual needs and preferences of patients, thereby maximising their efficacy and acceptance [37]. Also very important, to ensure that digital health solutions are accessible and usable for individuals with diverse impairments and abilities, developers need to adhere to the Web Content Accessibility Guidelines (WCAG) [50]. These guidelines foster user participation, access, and utilisation of digital solutions, which is particularly important as it ensures that technological advancements benefit all segments of the population [3].

While offering benefits, co-designing DTx have their difficulties with the most significant challenges being their complexity, resource constraints, and high costs. Co-creation requires a significant time investment, coordination among multiple stakeholders, and effective management of diverse perspectives, which can strain resources, particularly for small developers. Communication barriers, stakeholder vulnerabilities and diversity, subjectivity, and bias are also common obstacles in the co-creation process. Furthermore, the evolving nature of user preferences and behaviours means that designs must be continually adapted, adding an ongoing burden to the development cycle. Despite these challenges, a well-executed co-creation process can still provide considerable value, but it is crucial for stakeholders to carefully weigh the costs and time commitments involved [51,52].

#### 3.2.3. Examples of How Co-Creation Has Been Used in the Development of DTx

Although stakeholder involvement has rightly been given a central role in the development of a wide range of mHealth apps, there is a gap in studies on the use of participatory methodologies in the development of digital therapeutics.

GlucoseCoach—a diabetes self-management Tool [14]; WeCanManage—a self-management intervention for cancer survivors [13]; Actissist—an intervention grounded in cognitive behaviour therapy for early psychosis [10]; or even ValueCare, a study that employs co-design methodologies to understand users’ perspectives on a technological support solution for individuals who have experienced strokes, are good examples on the use of co-creation in mHealth [9].

On the other side, only two studies [15,16] and one piece of web-based content [17] have been found regarding co-creation in DTx. Concerning the approved DTx listed in Table 1, no evidence was found on the use of co-creation.

Barbaric, A. et al. present a co-design study guided by a literature review and user-centred design process to design, develop, and evaluate a voice app function for the previously existing smartphone-based heart failure programme called Medly, a medically prescribed DTx in Canada [15]. The DTx itself had undergone clinical trials demonstrating its efficacy, but a longitudinal study to assess adherence to the therapy revealed inconsistent results across different age groups and a decline in app usage over time. As older adults struggled with using the app, researchers concluded that it was necessary to explore the feasibility of designing a voice-based app to better serve this group of users.

In the first phase of the study, user needs were identified through a literature review, a market scan, and the existing Medly programme requirements, leading to the development of a prototype. In the second phase, user feedback was sought through usability studies—usability sessions, SUS questionnaires, and qualitative semi-structured interview sessions. The feedback collected was then incorporated and considered in the redesign of the app. According to the study results, most participants were satisfied with the design of the voice app, and almost all (88%) felt confident using it; however, when asked whether they would use it again, the most popular response was “neutral” [15].

While Barbaric et al.’s study effectively focuses on creating a voice-based app to assist older users, a notable limitation is the lack of direct user consultation during the initial design phase [15]. Relying primarily on literature reviews and secondary data may have resulted in a misalignment with actual user needs and challenges. Engaging users earlier in the design process could have provided valuable insights, potentially improving the app’s relevance and usability. This omission might explain the neutral feedback regarding users’ willingness to continue using the app, as it highlights a possible gap between the design and real user experiences. Time and participant limitations are also addressed in the study.

Gilson et al. present a study using co-design methodologies to develop Generation Connect, a digital therapeutic platform, designed to support caregivers of individuals with dementia [16]. The research involved conducting a series of focus groups with various stakeholders. This qualitative approach allowed the collection of comprehensive insights into the participants’ needs, barriers, and objectives. Five principal themes were identified: technology, care services, data documentation and outcomes, cost and finance, and resources for caregivers. These findings will guide the ongoing development of the platform to alleviate caregiving burdens, enhance cognitive and functional outcomes, and improve caregiver engagement. The subsequent phase entails assessing the platform’s effectiveness through a clinical trial to evaluate the efficacy of its evidence-based interventions and its market viability [16].

There is another example of the use of co-creation for developing DTx. The Digital Therapeutics for Diabetes project in Lebanon is an initiative launched by the Médecins Sans Frontières (MSF) Sweden Innovation Unit and the Operational Centre Geneva (OCG) in 2021 and is focused on developing a digital therapeutic solution for diabetes management [17]. The project, still under development, is prioritising people with lived experiences and clinicians as partners in the development, implementation, and evaluation process. Stakeholders are consistently engaged through interviews, surveys, focus groups, and workshops [53]. This continuous reliance on user feedback from the initial stages to the final phases of development exemplifies the effective use of co-creation methodologies in the application.

While it remains in the developmental stage and is not yet approved, it is notable that co-design methodologies are being implemented from the starting point. This proactive inclusion of co-design practises is pivotal for tailoring the platform to meet the actual needs and experiences of its users, potentially enhancing its efficacy and acceptance upon completion.

## 4. Conclusions

Digital health has grown and is transforming and revolutionising the healthcare sector, promoting accessibility and efficiency, and improving patients’ quality of life and overall well-being.

Digital therapeutics are health software intended to treat or alleviate a disease, disorder, condition, or injury by generating and delivering a medical intervention that has a demonstrable positive therapeutic impact on a patient’s health. The increase in funding for the development of these technologies and the growing number of regulatory bodies approving these solutions show that DTx are here to stay.

DTx bring many advantages, such as the possibility to enhance treatment accessibility; lower healthcare costs; the potential to provide insights into optimising patient services, ultimately leading to better health outcomes, like with remote monitoring, which allows health professionals to keep track of patient outcomes; deeper patient understanding of the disease/condition, greater involvement; self-management possibilities; accessibility; and personalised support.

Significant challenges include privacy and security concerns; the lack of a standardised evaluation and approval process for digital health products; a lack of user-friendliness; bugs; little integration into health systems; and little knowledge among health professionals.

With regulatory, usage, and safety improvements, it is expected that DTx will become more integrated into healthcare systems, which will require adjustments in the healthcare professions and in the system itself.

Including users in the design process is becoming the standard practise in health. Co-design is highly collaborative and can use techniques like ethnographic studies, usability studies, or workshops to fully integrate users’ perspectives into designs. However, the evidence on best practises for meaningful co-design with patients in health research is still lacking.

In this review, only three examples of the implementation of co-creation in DTx were found, which further demonstrates the lack of usage of these activities that can prove so advantageous for enhancing the adoption and adherence to digital therapeutics. The true potential of DTx will only be reached when patients are actively involved in all stages of development, evaluation, and use of these solutions.

This narrative review of the literature aims to provide valuable insights while acknowledging some limitations commonly associated with such reviews, including potential biases, a lack of a systematic methodology, and the inherent heterogeneity of the studies examined.

## 5. Future Directions and Recommendations

Future research will need to investigate the impact on patient safety and health outcomes when one or various DTx are used in combination. Such studies should assess the synergic effects, potential interactions, and overall efficacy of using one or multiple DTx. This will be crucial in understanding how combined DTx interventions can optimise therapeutic outcomes and enhance the overall quality of healthcare. Alongside security, privacy concerns must be at the top of the concerns of regulatory bodies.

Assessing user engagement and the usability of these platforms is extremely important if patients are to make effective use of them. The use of co-creation methodologies such as co-design and participatory design could help mitigate this.

With improved regulation pathways, it is expected that digital therapeutics become more integrated into healthcare systems, which will require adjustments in the healthcare professions and in the system itself. Providing ongoing training for healthcare professionals who want to learn more about the subject is of extreme importance, increasing acceptance and prescription. Some attention should also be given to providing training for patients and caregivers in the use of digital therapies.

In line with these concerns, Denecke et al. propose a DTx Risk Assessment Canvas, which could play a crucial role in identifying and assessing the risks and safety of digital therapeutics (DTx). This tool provides a structured framework for evaluating potential hazards and mitigating strategies, ensuring the safe and effective deployment of DTx in clinical practise. Despite its evident potential benefits, this scale still requires thorough evaluation. Nevertheless, it indicates a promising direction for future research and implementation to enhance the reliability and safety of DTx interventions [19].

Bochicchio et al., on the other hand, propose a collaborative method, involving patients and physicians, to assess the usability of digital therapeutics (DTx) software. The proposed method uses the International Measurement System (IMS) scale and the Mobile Application Rating Scale (MARS) in a framework based on a simplified version of phase III clinical trials [30].

Many of the reviewed studies exhibit limitations, primarily due to constraints related to lack of time, resources, and personnel. Recruiting participants poses further challenges, especially in studies involving human subjects and patients, which most of the time require the approval of ethics committees. One of the limitations identified is that most studies conduct only a single round of usability testing, which may not adequately capture the iterative improvements necessary for optimal user experience. Additionally, the sample sizes of user participants in these studies tend to be very small, limiting the generalisability of the findings. Although user involvement is typically acknowledged, it is often quite limited; participants are generally required to follow predefined procedures rather than engage actively in the design process. This approach can result in less effective and user-centric outcomes.

The design process should be an ongoing endeavour, instead of finishing once the DTx are developed. Continuous evaluation and refinement are essential to ensure that the apps remain relevant and effective over time. This iterative approach involves regularly gathering feedback from users, monitoring performance, and addressing emerging issues or evolving needs. By maintaining an active focus on improvement, designers can adapt to changes in user requirements, technological advancements, and market conditions.

## Figures and Tables

**Table 1:** List of evidence-based innovative DTx products currently available on the market.

Product Name	Classification	Company	Therapeutic Area	Approval Status	Website
Dario^®^ Platform	Non- Prescription DTx	Dario	Diabetes (type 1 and type 2) Hypertension	FDA-cleared Class II device; CE mark received by European Notified Body	https://www.dariohealth.com (accessed on 5 June 2024)
Insulia	Prescription DTx	Voluntis	Type 2 diabetes	FDA-510(k) EU-CE Mark	https://insulia.com/ (accessed on 5 June 2024)
Daylight^®^	Non- Prescription DTx	Big Health	Generalised anxiety disorder	EU-CE Mark FDA enforcement discretion	https://www.bighealth.com/daylight/ (accessed on 5 June 2024)
EndeavorOTC^®^	Non- Prescription DTx	Akili	Attention-deficit/ hyperactivity disorder (ADHD)	FDA-authorised Class II Medical Device	https://www.akiliinteractive.com/ (accessed on 5 June 2024))
Freespira^®^	Prescription DTx	Palo Alto Health Sciences	Post-traumatic stress disorder (PTSD), panic disorder, panic/anxiety attacks	FDA-cleared Class II Medical Device	https://freespira.com/ (accessed on 5 June 2024)
GameChange^®^	Non- Prescription DTx	RealizedCare	Agoraphobic avoidance and distress	Class I CE mark	https://www.realizedcare.com/ (accessed on 5 June 2024)
HelloBetter^®^ Chronic Pain	Non- Prescription DTx (Eligible in ‘DiGA’)	HelloBetter	Chronic Pain	Class I CE mark (MDR)	https://hellobetter.de/en/ (accessed on 5 June 2024)
HelloBetter^®^ Diabetes	Non- Prescription DTx (Eligible in ‘DiGA’)	HelloBetter	Diabetes	Class I CE mark (MDD)	https://hellobetter.de/en/ (accessed on 5 June 2024)
HelloBetter^®^ Panic	Non- Prescription DTx (Eligible in ‘DiGA’)	HelloBetter	Panic disorder with or without agoraphobia	Class I CE mark (MDR)	https://hellobetter.de/en/ (accessed on 5 June 2024)
HelloBetter^®^ Sleep	Non- Prescription DTx (Eligible in ‘DiGA’)	HelloBetter	Insomnia	Class I CE mark (MDR)	https://hellobetter.de/en/ (accessed on 5 June 2024)
HelloBetter^®^ Stress and Burnout	Non- Prescription DTx (Eligible in ‘DiGA’)	HelloBetter	Stress and burnout	Class I CE mark (MDR)	https://hellobetter.de/en/ (accessed on 5 June 2024)
HelloBetter^®^ Vaginismus Plus	Non- Prescription DTx (Eligible in ‘DiGA’)	HelloBetter	Vaginismus, dyspareunia, and genito-pelvic pain/penetration disorder	Class I CE mark (MDR)	https://hellobetter.de/en/ (accessed on 5 June 2024)
JOGO-Gx^®^	Non- Prescription DTx (Reimbursed by Medicare)	Jogo Health	Migraine and chronic lower back pain	FDA 510K exempted (reviewed by the FDA and registered)	https://www.jogohealth.com/ (accessed on 5 June 2024)
Leva^®^	Non- Prescription DTx	Leva	Urinary and faecal incontinence	FDA-cleared Class II Medical Device	https://www.levatherapy.com/ (accessed on 5 June 2024)
Propeller^®^	Non- Prescription DTx	ResMed	Asthma and chronic obstructive pulmonary disease (COPD)	FDA-cleared Class II Medical Device and EU Class I Medical Device	https://propellerhealth.com/ (accessed on 5 June 2024)
Sleepio^®^	Non- Prescription DTx	Big Health	Insomnia	FDA enforcement discretion; Class I CE mark	https://www.bighealth.com/sleepio/ (accessed on 5 June 2024)
Welldoc^®^ App	Non- Prescription DTx	Welldoc	Type 1 and 2 diabetes, pre-diabetes, hypertension, heart failure, weight and obesity management	FDA-cleared Class II Medical Device; Class IIa CE mark; Health Canada-licenced Class II Medical Device	https://www.welldoc.com/ (accessed on 5 June 2024)
reSET^®^	Prescription DTx	Pear Therapeutics	Substance-use disorder (SUD)	FDA-cleared Class II Medical Device	https://peartherapeutics.com/products/reset-reset-o/ (accessed on 5 June 2024)
Natural Cycles	-	Natural Cycles	Birth control	FDA-de novo	https://www.naturalcycles.com (accessed on 5 June 2024)
Oleena	Prescription DTx	Voluntis	All cancer	FDA-510(k)	https://oleena.com/ (accessed on 5 June 2024)
CureApp-SC	Non- Prescription DTx	CuraApp Inc.	Smoking cessation	MHLW (Japan)	https://sc.cureapp.com/d/ (accessed on 5 June 2024)
PainChek^®^	Non-Prescription DT	PainChek	Pain in people living with dementia	Class I CE Mark	https://www.painchek.com/ (accessed on 5 June 2024)

## References

[1] Adler R.F., Baez K., Morales P., Sotelo J., Victorson D., Magasi S. (2024). Evaluating the Usability of an mHealth App for Empowering Cancer Survivors With Disabilities: Heuristic Evaluation and Usability Testing. JMIR Hum. Factors.

[2] Dang A., Arora D., Rane P. (2020). Role of digital therapeutics and the changing future of healthcare. J. Fam. Med. Prim. Care.

[3] Henni S.H., Maurud S., Fuglerud K.S., Moen A. (2022). The experiences, needs and barriers of people with impairments related to usability and accessibility of digital health solutions, levels of involvement in the design process and strategies for participatory and universal design: A scoping review. BMC Public Health.

[4] Petracca F., Ciani O., Cucciniello M., Tarricone R. (2020). Harnessing Digital Health Technologies During and After the COVID-19 Pandemic: Context Matters. J. Med. Internet Res..

[5] Warraich H.J., Califf R.M., Krumholz H.M. (2018). The digital transformation of medicine can revitalize the patient-clinician relationship. NPJ Digit. Med..

[6] Farnood A., Johnston B., Mair F.S. (2020). A mixed methods systematic review of the effects of patient online self-diagnosing in the ‘smart-phone society’ on the healthcare professional-patient relationship and medical authority. BMC Med. Inform. Decis. Mak..

[7] DTA (2024). Product Library. https://dtxalliance.org/understanding-dtx/product-library/.

[8] Lofton J.C. (2020). AMCP Partnership Forum: Digital Therapeutics-What Are They and Where Do They Fit in Pharmacy and Medical Benefits?. J. Manag. Care Spec. Pharm..

[9] Bally E.L.S., Cheng D., van Grieken A., Ferri Sanz M., Zanutto O., Carroll A., Darley A., Roozenbeek B., Dippel D.W.J., Raat H. (2023). Patients’ Perspectives Regarding Digital Health Technology to Support Self-management and Improve Integrated Stroke Care: Qualitative Interview Study. J. Med. Internet Res..

[10] Berry N., Machin M., Ainsworth J., Berry K., Edge D., Haddock G., Lewis S., Morris R., Bucci S. (2020). Developing a Theory-Informed Smartphone App for Early Psychosis: Learning Points From a Multidisciplinary Collaboration. Front. Psychiatry.

[11] Bevan Jones R., Stallard P., Agha S.S., Rice S., Werner-Seidler A., Stasiak K., Kahn J., Simpson S.A., Alvarez-Jimenez M., Rice F. (2020). Practitioner review: Co-design of digital mental health technologies with children and young people. J. Child Psychol. Psychiatry Allied Discip..

[12] Lee N.J., Ahn S., Lee M. (2020). Mixed-method investigation of health consumers’ perception and experience of participation in patient safety activities. BMJ Open.

[13] Adler R.F., Morales P., Sotelo J., Magasi S. (2022). Developing an mHealth App for Empowering Cancer Survivors with Disabilities: Co-design Study. JMIR Form. Res..

[14] Bonet-Olivencia S., Carrillo-Leal J., Rao A., Sasangohar F. (2024). User-Centered Design of a Diabetes Self-Management Tool for Underserved Populations. J. Diabetes Sci. Technol..

[15] Barbaric A., Munteanu C., Ross H., Cafazzo J.A. (2022). Design of a Patient Voice App Experience for Heart Failure Management: Usability Study. JMIR Form. Res..

[16] Gilson A., Gassman M., Dodds D., Lombardo R., Ford Ii J.H., Potteiger M. (2022). Refining a Digital Therapeutic Platform for Home Care Agencies in Dementia Care to Elicit Stakeholder Feedback: Focus Group Study With Stakeholders. JMIR Aging.

[17] MSF Sweden Innovation Unit (2023). Digital Therapeutics (DTx) for Diabetes. https://msf-siu.org/development-stage-cases/digital-therapeutics-for-diabetes.

[18] DTA (2024). What is a DTx?. https://dtxalliance.org/understanding-dtx/.

[19] Denecke K., May R., Gabarron E., Lopez-Campos G.H. (2023). Assessing the Potential Risks of Digital Therapeutics (DTX): The DTX Risk Assessment Canvas. J. Pers. Med..

[20] DTA (2022). Is This Product a DTx?. https://dtxalliance.org/understanding-dtx/what-is-a-dtx/#difference.

[21] Hong J.S., Wasden C., Han D.H. (2021). Introduction of digital therapeutics. Comput. Methods Programs Biomed..

[22] Brönneke J.B., Herr A., Reif S., Stern A.D. (2023). Dynamic HTA for digital health solutions: Opportunities and challenges for patient-centered evaluation. Int. J. Technol. Assess. Health Care.

[23] FDA (2018). Software as a Medical Device (SaMD). https://www.fda.gov/medical-devices/digital-health-center-excellence/software-medical-device-samd.

[24] Huh K.Y., Oh J., Lee S., Yu K.S. (2022). Clinical Evaluation of Digital Therapeutics: Present and Future. Healthc. Inform. Res..

[25] Palanica A., Docktor M.J., Lieberman M., Fossat Y. (2020). The Need for Artificial Intelligence in Digital Therapeutics. Digit. Biomark..

[26] Cornet V.P., Daley C., Bolchini D., Toscos T., Mirro M.J., Holden R.J. (2019). Patient-centered Design Grounded in User and Clinical Realities: Towards Valid Digital Health. Proc. Int. Symp. Hum. Factors Ergon. Health Care.

[27] Abdulhussein F.S., Pinkney S., Görges M., van Rooij T., Amed S. (2023). Designing a Collaborative Patient-Centered Digital Health Platform for Pediatric Diabetes Care in British Columbia: Formative Needs Assessment by Caregivers of Children and Youths Living With Type 1 Diabetes and Health Care Providers. JMIR Pediatr. Parent..

[28] Ahlqvist J., Kalliola M. (2021). How Can Digital Therapeutics Help Europe?.

[29] European Union (2024). Digital Therapeutics (Dtx). https://www.edps.europa.eu/press-publications/publications/techsonar/digital-therapeutics-dtx.

[30] Bochicchio M.A., Vaira L., Mortara A., Maria R.D. Which Usability Assessment for Digital Therapeutics and Patient Support Programs?. Proceedings of the 2021 IEEE International Conference on Digital Health (ICDH).

[31] Patel N.A., Butte A.J. (2020). Characteristics and challenges of the clinical pipeline of digital therapeutics. NPJ Digit. Med..

[32] Yao R., Zhang W., Evans R., Cao G., Rui T., Shen L. (2022). Inequities in Health Care Services Caused by the Adoption of Digital Health Technologies: Scoping Review. J. Med. Internet Res..

[33] Gan D.Z.Q., McGillivray L., Larsen M.E., Torok M. (2023). Promoting engagement with self-guided digital therapeutics for mental health: Insights from a cross-sectional survey of end-users. J. Clin. Psychol..

[34] Kim J., Park K.M., Lee S., Park S., Hong M., Shin J., Lee E. (2024). Understanding patient perspectives on digital therapeutics and its platform for insomnia: Insights from focused group interviews. BMC Health Serv. Res..

[35] Carrera A., Lettieri E., Lietti G., Martignoni S., Sgarbossa C., Cafazzo J. (2024). Therapies go digital. What drives physicians’ acceptance?. PLoS ONE.

[36] Laurisz N., Ćwiklicki M., Żabiński M., Canestrino R., Magliocca P. (2023). Co-Creation in Health 4.0 as a New Solution for a New Era. Healthcare.

[37] Carrera A., Manetti S., Lettieri E. (2024). Rewiring care delivery through Digital Therapeutics (DTx): A machine learning-enhanced assessment and development (M-LEAD) framework. BMC Health Serv. Res..

[38] Barros J.P., Brandão P. End-Stage Renal Disease Self-management: Mobile app development. Proceedings of the 2021 IEEE Symposium on Computers and Communications (ISCC).

[39] Denecke K., Von Kaenel F., Miletic M., Fernández-Llatas C., Ibañez-Sánchez G., Valero-Ramón Z., Martînez-Millana A., Segura M., Rivera Romero O. (2023). How to Design Successful Participatory Design Workshops for Digital Health Solutions?. Caring is Sharing—Exploiting the Value in Data for Health and Innovation.

[40] Slattery P., Saeri A.K., Bragge P. (2020). Research co-design in health: A rapid overview of reviews. Health Res. Policy Syst..

[41] SDT (2024). User Scenarios. https://servicedesigntools.org/tools/user-scenarios.

[42] SDT (2024). Personas. https://servicedesigntools.org/tools/personas.

[43] Muratovski G., Steele M. (2016). Research for Designers.

[44] Moran K. (2019). Usability Testing 101. https://www.nngroup.com/articles/usability-testing-101/.

[45] Borycki E.M., Kushniruk A.W. (2022). Health technology, quality and safety in a learning health system. Healthc. Manag. Forum.

[46] Martin G., Arora S., Shah N., King D., Darzi A. (2020). A regulatory perspective on the influence of health information technology on organisational quality and safety in England. Health Inform. J..

[47] Ebnali M., Kennedy-Metz L.R., Conboy H.M., Clarke L.A., Osterweil L.J., Avrunin G., Miccile C., Arshanskiy M., Phillips A., Zenati M.A. (2022). A Coding Framework for Usability Evaluation of Digital Health Technologies. Human-Computer Interaction. Theoretical Approaches and Design Methods.

[48] Jeong H., Yoo J.H., Goh M. Virtual Agents in Internet-Based Cognitive Behavioral Therapy: Enhancing Engagement and Alleviating Depression. Proceedings of the 2023 IEEE International Conference on Agents (ICA).

[49] Grannell A., Hallson H., Gunlaugsson B., Jonsson H. (2023). Exercise therapy as a digital therapeutic for chronic disease management: Consideration for clinical product development. Front. Digit. Health.

[50] W3C (2023). Web Content Accessibility Guidelines (WCAG) 2.1. https://www.w3.org/TR/WCAG21/.

[51] Noorbergen T.J., Adam M.T.P., Teubner T., Collins C.E. (2021). Using Co-design in Mobile Health System Development: A Qualitative Study with Experts in Co-design and Mobile Health System Development. JMIR Mhealth Uhealth.

[52] Papoutsi C., Wherton J., Shaw S., Morrison C., Greenhalgh T. (2021). Putting the social back into sociotechnical: Case studies of co-design in digital health. J. Am. Med. Inform. Assoc..

[53] MSF Sweden Innovation Unit (2023). Prioritizing People with Lived Experience as Partners to Co-Create a Diabetes Digital Therapeutic (DTx) in Lebanon. https://msf-siu.org/blog/co-creating-a-diabetes-digital-therapeutic-in-lebanon.

